# Testing of a Tool for Prostate Cancer Screening Discussions in Primary Care

**DOI:** 10.3389/fonc.2018.00238

**Published:** 2018-06-28

**Authors:** Anita D. Misra-Hebert, Grant Hom, Eric A. Klein, Janine M. Bauman, Niyati Gupta, Xinge Ji, Andrew J. Stephenson, J. Stephen Jones, Michael W. Kattan

**Affiliations:** ^1^Department of Internal Medicine, Cleveland Clinic, Cleveland, OH, United States; ^2^Center for Value-Based Care Research, Cleveland Clinic, Cleveland, OH, United States; ^3^Department of Quantitative Health Sciences, Cleveland Clinic, Cleveland, OH, United States; ^4^Case Western Reserve University, Cleveland, OH, United States; ^5^Glickman Urological and Kidney Institute, Cleveland Clinic, Cleveland, OH, United States

**Keywords:** prostate cancer, cancer screening, shared decision-making, prostate-specific antigen, clinical decision-making

## Abstract

**Background:**

As prostate cancer (PCa) screening decisions often occur in outpatient primary care, a brief tool to help the PCa screening conversation in busy clinic settings is needed.

**Methods:**

A previously created 9-item tool to aid PCa screening discussions was tested in five diverse primary care clinics. Fifteen providers were recruited to use the tool for 4 weeks, and the tool was revised based upon feedback. The providers then used the tool with a convenience sample of patients during routine clinic visits. Pre- and post-visit surveys were administered to assess patients’ knowledge of the option to be screened for PCa and of specific factors to consider in the decision. McNemar’s and Stuart–Maxwell tests were used to compare pre-and post-survey responses.

**Results:**

14 of 15 providers completed feedback surveys and had positive responses to the tool. All 15 providers then tested the tool on 95 men aged 40–69 at the five clinics with 2–10 patients each. The proportion of patients who strongly agreed that they had the option to choose to screen for PCa increased from 57 to 72% (*p* = 0.018) from the pre- to post-survey, that there are factors in the personal or family history that may affect PCa risk from 34 to 47% (*p* = 0.012), and that their opinions about possible side effects of treatment for PCa should be considered in the decision from 47 to 61% (*p* = 0.009).

**Conclusion:**

A brief conversation tool for the PCa screening discussion was well received in busy primary-care settings and improved patients’ knowledge about the screening decision.

## Introduction

The current US Preventive Services Task Force (USPSTF) Prostate Cancer (PCa) Screening Recommendations for men aged 55–69 suggest individualized decision-making for the PCa screening decision after a discussion with a clinician of benefits, harms, and consideration of a patient’s values and preferences (1). For younger men, the previous American Urological Association guideline also suggests individualized screening decisions for men aged 40–54 at increased risk with a family history of PCa or for African-American men (2). Controversy remains regarding optimal PCa screening strategies (3) and the ability to engage in these conversations in primary care settings (4) where many PCa screening conversations may occur. We previously created a 9-item brief tool for PCa screening conversations (5). The goal of the tool is to aid PCa screening discussions where the patient and provider can discuss the risks and benefits of PCa screening given a patient’s individual risk factor, health status, and preferences. We tested this tool with primary care providers and patients to determine its ease of use for providers and patients and to describe the responses in primary care settings.

## Materials and Methods

Each item on the PCa conversation tool is scored from 0 to 3 (minimum score 0, maximum score 27) with higher scores suggesting PCa screening may be beneficial. We tested the tool in three steps in five diverse urban and suburban primary care clinics. First, 15 primary care providers were recruited using an electronic mail announcement about the study to agree to use the tool for 4 weeks from June to July 2017 with up to 10 patients each. At the end of the 4 weeks, to assess provider experience with the tool, the validated Perceived Usefulness (6-item) and Perceived Ease of Use (6-item) scales (6) were sent electronically to providers through REDCap (7). The 12 questions on these two scales have seven options ranging from extremely likely to extremely unlikely. The conversation tool was revised based upon feedback and a final version was created (Appendix S1 in Supplementary Material). In the last step, the same group of 15 providers was asked to use the tool with a convenience sample of patients during routine clinic visits from September to December 2017. A member of the research team reviewed provider schedules for eligible male patients aged 40–69 who had no prior history of PCa. If the provider and patient agreed to participate in the study at the time of the appointment, a 4-item pre-visit survey was administered to the patient by a research assistant. The provider then used the conversation tool with the patient. Previously created reference information that was available through the electronic medical record to all of the participating primary care providers regarding PCa screening, thus part of usual care, was made available to the provider in hard copy to use during the visit. After the visit, a 5-item post-visit survey was administered. These surveys had questions related to patients’ knowledge that they have an option to be screened for PCa and of specific factors to be considered in the decision. Each question was scored on a 5-point Likert scale with a range of strongly disagree (1) to strongly agree (5) (Appendix S2 and S3 in Supplementary Material). McNemar’s test was used to compare pre-and post-survey strongly-agree vs other responses. Stuart–Maxwell test was used to compare marginal homogeneity for all pre-and post-survey responses. This protocol was approved by the Institutional Review Board at Cleveland Clinic.

## Results

**Figure 1:**
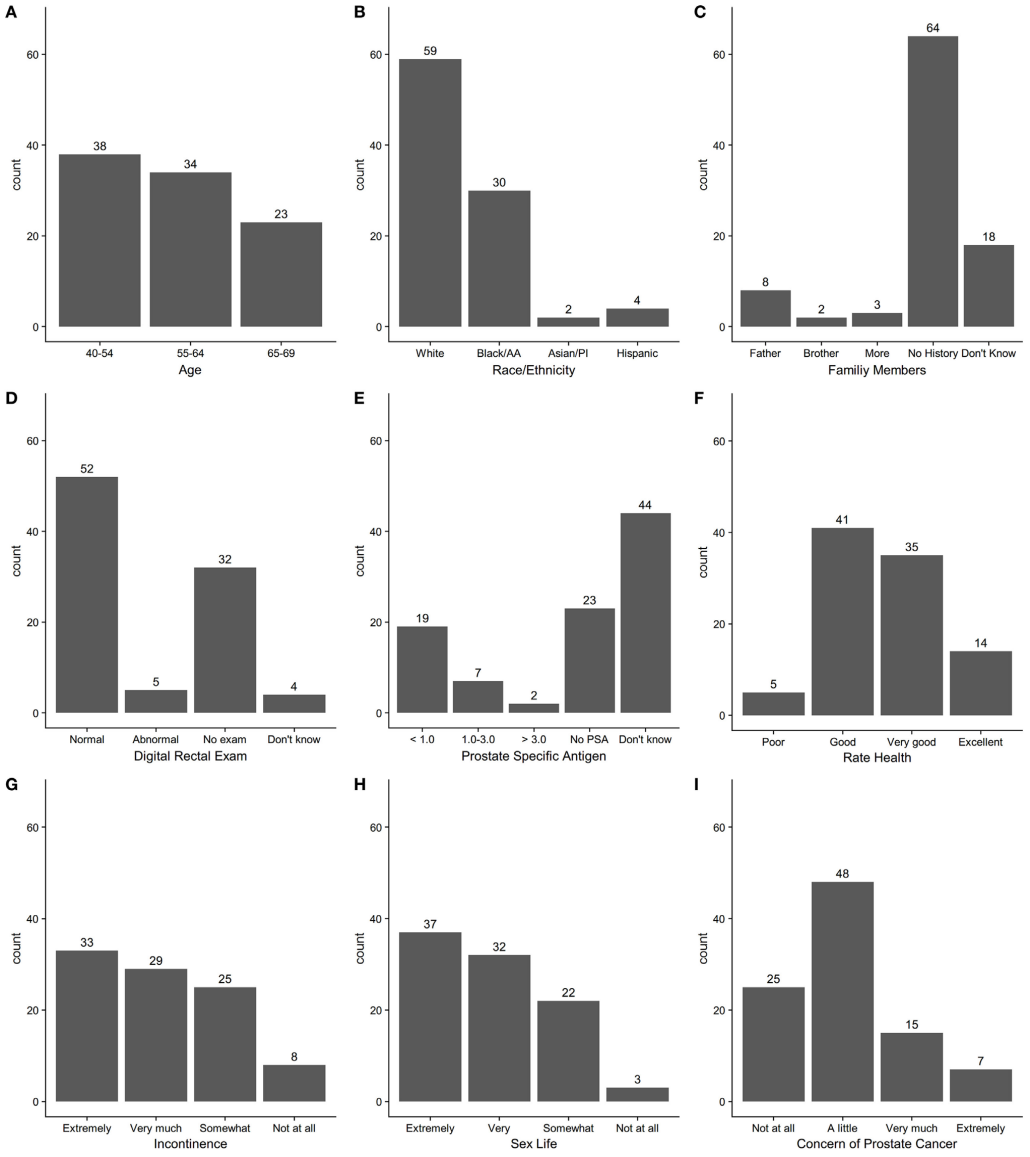

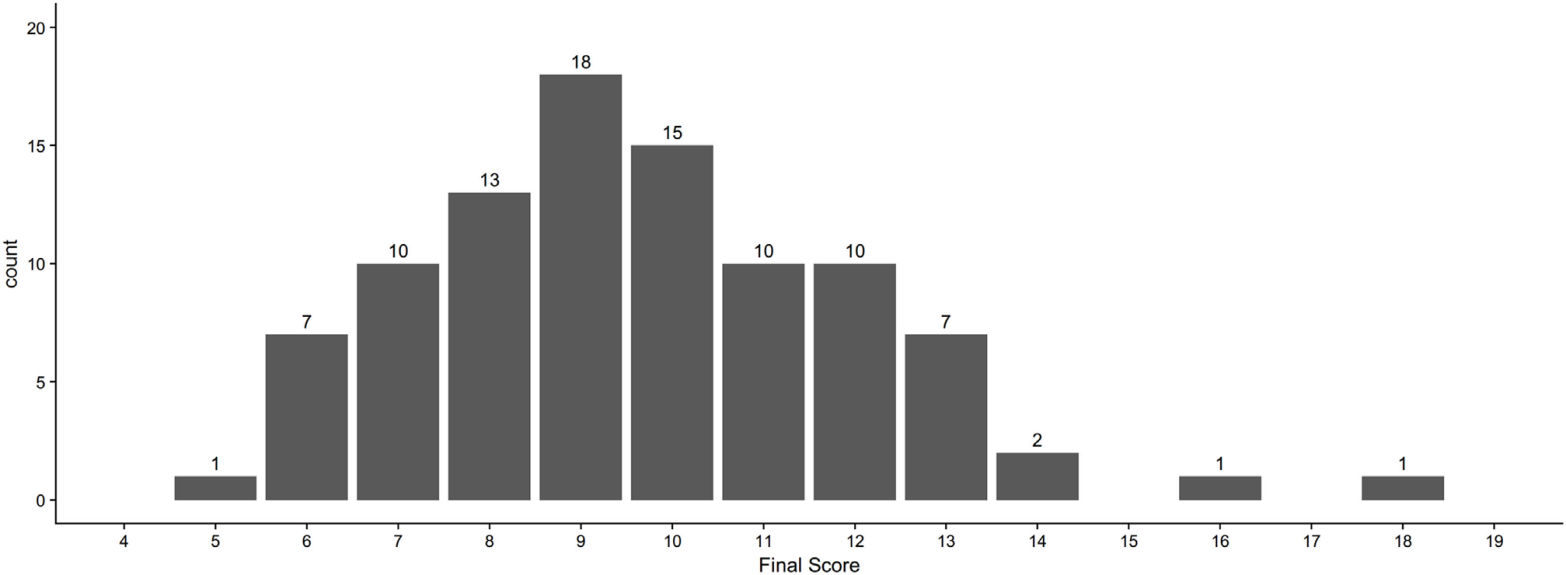
Count distribution of individual question responses for questions 1–9 **(A–I)** and total score on prostate cancer screening conversation tool.

After the first step, 14 of 15 providers completed the Perceived Usefulness and Perceived Ease of Use scales (6). Results of responses to the survey questions are shown in Appendix S4 in Supplementary Material. Providers had overall positive responses that the tool would “enhance my effectiveness (64%)” be “clear and understandable (79%),” and “easy to use (86%).” The 15 providers tested the final version of the tool on 95 patients aged 40–69 at the five clinics with 2–10 patients per provider. Patient participants included 40% aged 40–54, 36% aged 55–64, and 24% aged 65–69, and were 62% Caucasian, 32% Black, 2% Asian, and 4% Hispanic. The distribution of scores for each question and total score on the PCa conversation tool are shown in Figure 1. Most patients had total scores in the range of 7–12 on the instrument. Comparison of the pre- and post-visit survey responses are shown in Table 1. Further analysis of the proportion of patients who strongly agreed that they had the option to choose to screen for PCa increased from 57 to 72% (*p* = 0.018) from the pre- to post-survey, that there are factors in the personal or family history that may affect risk for PCa from 34 to 47% (*p* = 0.012), and that their opinions about possible side effects of treatment for PCa should be considered in the decision from 47 to 61% (*p* = 0.009). When selecting from a list, the three most important factors in the PCa screening decision, the most frequent responses were age (65%), family history of PCa (42%), and concern about developing PCa (36%). Concern about sex life and leakage of urine were among the three most important factors for 10 and 18% of patients, respectively (Table 1). For the question of choosing the three most important factors in the PCa screening decision, we also compared responses of patients age <55 to those ≥55 years and found no significant differences in frequency of responses in the two age groups (results not shown).

**Table 1:** Survey responses with use of prostate cancer (PCa) screening conversation tool.

Question: I have an option to choose the prostate-specific antigen (PSA) blood test to screen for PCa: responses *N* (%)			

	Pre-survey	Post-survey	*p*
1 Strongly disagree	2 (2%)	3 (3%)	0.075
2	2 (2%)	1 (1%)	
3	17 (18%)	8 (8%)	
4	20 (21%)	15 (16%)	
5 Strongly agree	54 (57%)	68 (72%)	

**Question: there are factors in my personal or family history that may affect my risk for developing PCa: response *N* (%)**			
1 Strongly disagree	12 (13%)	7 (7%)	0.014
2	8 (8%)	11 (12%)	
3	23 (24%)	16 (17%)	
4	20 (21%)	16 (17%)	
5 Strongly agree	32 (34%)	45 (47%)	

**Question: my opinions about my current health status should be considered in my decision to be screened for PCa: response *N* (%)**			
1 Strongly disagree	3 (3%)	4 (4%)	0.171
2	9 (10%)	3 (3%)	
3	13 (14%)	14 (15%)	
4	16 (17%)	24 (25%)	
5 Strongly agree	54 (57%)	50 (53%)	

**Question: my opinions about possible side effects of treatment for PCa should be considered in my decision to be screened for PCa: response *N* (%)**			
1 Strongly disagree	6 (6%)	4 (4%)	0.007
2	7 (7%)	4 (4%)	
3	14 (15%)	6 (6%)	
4	23 (24%)	23 (24%)	
5 Strongly agree	45 (47%)	58 (61%)	

**Question: most important factors in decision to accept or reject PCa screening with the PSA test: post-visit survey responses**			

**Reason**		**Post-survey: frequency**	
My age		62 (65%)	
My family history of PCa		40 (42%)	
My concern about developing PCa		34 (36%)	
Previous or current physical exam		27 (28%)	
My opinion about how healthy I am overall		27 (28%)	
Previous or current PSA test result		25 (26%)	
My provider’s recommendation		25 (26%)	
My concern about leakage of urine		17 (18%)	
My race or ethnicity		14 (15%)	
My concern about sex life		9 (10%)	

## Discussion

Our study demonstrated the feasibility of using a PCa screening conversation tool in busy primary-care settings to address important aspects of the PCa screening discussion. Providers found the tool easy to use and after the tool was used during the visit, patients were more likely to strongly agree that they had an option to screen for PCa, that there were factors in their personal and family history that may affect their risk for PCa, and that their opinions about possible side effects of treatment for PCa should be considered in the screening decision. As very few patients scored very low or high on the tool, responses did not readily direct the patient to a decision that screening would or would not be preferred. However, the tool effectively introduced the screening conversation.

Discussions about PCa screening may not be of high quality (8) and cancer screening guidelines may not always provide the optimal level of information to aid these discussions (9). Allowing patients to understand that they have an option to make a decision is a key component of operationalizing shared decision-making in a clinical setting (10). Decision aids for PCa screening can improve patient knowledge and involvement in decision making (11) and the interaction with a provider in addition to decision aid use alone may improve patient understanding of the PCa screening decision (12). The use of our tool prompted providers to cover the key domains that are relevant to a PCa screening decision and identify those men who could benefit from more extensive shared decision-making conversations if they scored in the mid-range on the tool.

Interestingly, when asked post-visit, patients most frequently chose their age, family history, and concern about developing PCa as important to the screening decision. Concern about leakage of urine or sex life were cited less frequently, although these are the most common potential downstream effects of PCa treatment that are often discussed. Our findings highlight that understanding and addressing patients’ anxiety about the PCa diagnosis should be an important component of the conversation, especially as related to ability to accept no immediate treatment for low-risk disease (13), given the option of active surveillance in this situation. While providers had access to reference information regarding population risks and benefits of PCa screening, decision aids providing individualized risk prediction of PCa screening outcomes, including the likelihood of additional downstream testing, may further improve the quality of the screening discussions, as patients often do not understand harms of screening tests (14) but rather focus on benefits.

A limitation of our study is that 40% of patients enrolled were in the younger age group of 40–54, thus our summary findings may not fully reflect the views of men in the age group (55–69) that the current USPSTF recommends for individualized decision-making for PCa screening. However, we believe our findings across these age groups remain relevant to practicing clinicians.

## Conclusion

We demonstrated the usefulness of a brief PCa conversation tool in primary care settings to improve PCa screening conversations and to identify the need for further shared decision-making around the PCa screening decision. Future work will focus on the implementation of the PCa tool in additional settings and on the outcome of the screening decision after use of the tool.

## Ethics Statement

This protocol was approved by the Institutional Review Board at Cleveland Clinic. All patient participants gave verbal consent to participation in the study after discussion of the Institutional Review Board—approved study information sheet.

## Author Contributions

AM-H designed the protocol, supervised the data collection and analysis, and drafted and revised the final manuscript. GH, EK, J-SJ, JB, XJ, and MK contributed to protocol development. GH administered the patient surveys and created the databases for the study information. NG administered the patient surveys. XJ performed the data analysis. AS reviewed the data and manuscript and made substantial contributed to revisions. All authors reviewed the final draft of the manuscript for important intellectual content.

## Conflict of Interest Statement

MK reports research funding from Novo Nordisk, Otsuka, Celgene, and Merck. He is a consultant for Novartis. AM-H, XJ, and JB report receiving research funding from Novo Nordisk and Merck. The remaining coauthors declare that the research was conducted in the absence of any commercial or financial relationships that could be construed as a potential conflict of interest.

## References

[1] US Preventive Services Task Force Grossman DC Curry SJ Owens DK Bibbins-Domingo K Caughey AB Screening for prostate cancer: US preventive services task force recommendation statement. JAMA (2018) 319(18):1901–13. 10.1001/jama.2018.3710 29801017

[2] Carter HB Albertsen PC Barry MJ Etzioni R Freedland SJ Greene KL Early detection of prostate cancer: AUA guideline. J Urol (2013) 190(2):419–26. 10.1016/j.juro.2013.04.119 23659877 PMC4020420

[3] Faiena I Holden S Cooperberg MR Soule HR Simons JW Morgan TM Prostate cancer screening and the goldilocks principle: how much is just right? J Clin Oncol (2018) 36(10):937–41. 10.1200/JCO.2017.76.4050 29401003 PMC6804825

[4] Mathew P Hachem H Han P Navigating prostate cancer screening in the real world of primary care: the mirage and the quicksand. JAMA Oncol (2018) 4(4):453–4. 10.1001/jamaoncol.2017.5682 29470576

[5] Misra-Hebert AD Kattan MW Prostate cancer screening: a brief tool to incorporate patient preferences in a clinical encounter. Front Oncol (2016) 6:235 10.3389/fonc.2016.00235 27867909 PMC5095121

[6] Davis FD Perceived usefulness, perceived ease of use, and user acceptance of information technology. MIS Q (1989) 13(3):319–40. 10.2307/249008

[7] Harris PA Taylor R Thielke R Payne J Gonzalez N Conde JG Research electronic data capture (REDCap)—A metadata-driven methodology and workflow process for providing translational research informatics support. J Biomed Inform (2009) 42(2):377–81. 10.1016/j.jbi.2008.08.010 18929686 PMC2700030

[8] Turini GA Gjelsvik A Renzulli JF. The state of prescreening discussions about prostate-specific antigen testing following implementation of the 2012 United States Preventive Services Task Force Statement. Urology (2017) 104:122–30. 10.1016/j.urology.2016.12.069 28322897

[9] Caverly TJ Hayward RA Reamer E Zikmund-Fisher BJ Connochie D Heisler M Presentation of benefits and harms in US cancer screening and prevention guidelines: systematic review. J Natl Cancer Inst (2016) 108(6):djv436. 10.1093/jnci/djv436 26917630 PMC5009951

[10] Elwyn G Frosch D Thomson R Joseph-Williams N Lloyd A Kinnersley P Shared decision making: a model for clinical practice. J Gen Intern Med (2012) 27(10):1361–7. 10.1007/s11606-012-2077-6 22618581 PMC3445676

[11] Volk RJ Hawley ST Kneuper S Holden EW Stroud LA Cooper CP Trials of decision aids for prostate cancer screening: a systematic review. Am J Prev Med (2007) 33(5):428–34. 10.1016/j.amepre.2007.07.030 17950409

[12] Stamm AW Banerji JS Wolff EM Slee A Akapame S Dahl K A decision aid versus shared decision making for prostate cancer screening: results of a randomized, controlled trial. Can J Urol (2017) 24(4):8910–7. 28832310

[13] Vickers AJ Edwards K Cooperberg MR Mushlin AI A simple schema for informed decision making about prostate cancer screening. Ann Intern Med (2014) 161(6):441 10.7326/M14-0151 25222389 PMC4412472

[14] Sutkowi-Hemstreet A Vu M Harris R Brewer NT Dolor RJ Sheridan SL Adult patients’ perspectives on the benefits and harms of overused screening tests: a qualitative study. J Gen Intern Med (2015) 30(11):1618–26. 10.1007/s11606-015-3283-9 25869017 PMC4617933

