# Supplementary material for: Testing of a Tool for Prostate Cancer Screening Discussions in Primary Care
Source: Front Oncol. 2018 Jun 28;8:238. doi: 10.3389/fonc.2018.00238 (PMC6031706; doi:10.3389/fonc.2018.00238)

Appendix S1

**Prostate Cancer Screening Tool**

| **Question** | **Answer Options with suggested scoring** | |
| --- | --- | --- |
| 1. What is your age? | ≥ 70 (0)  65-69 (3)  55-64 (2)  40-54 (1)  <40 (0) | |
| 2. What is your race or ethnicity? Select all that apply | White/Caucasian (1)  Black/African-American (3)  Asian/Pacific Islander (0)  Native American/Alaskan Native (0)  Hispanic (0) | |
| 3. Did any of your family members have prostate cancer? More than one answer may apply. | My father had prostate cancer (1)  My brother had prostate cancer (2)  More than one family member had prostate cancer (1)  (*Family members related by birth, not by marriage only. Skip this question if both father and brother had prostate cancer*)  I do not have a family history of prostate cancer(0)  I don’t know about my family history of prostate cancer (0) | |
| 4. If you have had a digital rectal exam, what was the result? | I don’t know the results (1)  No previous digital rectal exam (1)  Normal digital rectal exam (0)  Abnormal digital rectal exam (3) | |
| 5. If you have had previous prostate-specific antigen (PSA) level what was the result? | I don’t know (1)  No previous PSA (1)  PSA <1.0 (0)  PSA 1.0-3.0 (2)  PSA > 3.0 (3) | |
| 6. How would you rate your heath? | My health is excellent (3)  My health is very good (2)  My health is good (1)  My health is poor (0) | |
| 7. If you were to have problems with incontinence (leakage) of urine, how much would this bother you? | Not at all (3)  Somewhat (2)  Very much (1)  Extremely (0) | |
| 8. How important is your sex life and maintaining ability to have an erection? | Not important (3)  Somewhat important (2)  Very Important (1)  Extremely important (0) | |
| 9. Are you concerned about having or developing prostate cancer? | | No, I am *not concerned* about having or developing prostate cancer (0)  I am a *little concerned* I may have or may develop prostate cancer (1)  I am *very concerned* I may have or may develop prostate cancer (2)  I am *extremely concerned* I may have or may develop prostate cancer (3) |


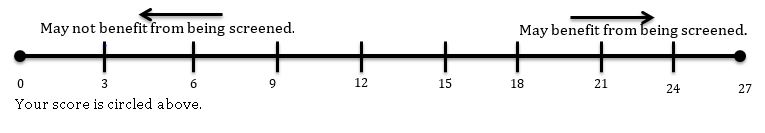
**For Office Use Only:**

| **Yes, you may benefit from being screened with the PSA if higher scores:** | **No, you may not benefit from being screened with the PSA if lower scores:** |
| --- | --- |
| Younger age, treating prostate cancer may have more benefits than risks  Extensive family history of prostate cancer  Higher risk racial group  Abnormal digital rectal exam or no previous digital rectal exam  Previous PSA in higher risk range or no Previous PSA  Excellent health status, life expectancy not reduced related to comorbid conditions  Urinary incontinence would not be bothersome  Sex life is not important  Extremely concerned about having or developing prostate cancer | Older age, treating prostate cancer may have more risks than benefits  No family history of prostate cancer  Lower risk racial group  Normal previous digital rectal exam  Previous PSA in lower risk range  Poor health status, life expectancy reduced related to comorbid conditions  Urinary incontinence would be extremely bothersome  Sex life is extremely important  Not concerned about having or developing prostate cancer |

Appendix S2

Patient Survey-Pre

1. I have an OPTION to choose the PSA (prostate specific antigen) blood test to screen for prostate cancer.

1 2 3 4 5

Strongly Disagree Strongly Agree

1. There are factors in my personal or family history that may affect my risk for developing prostate cancer.

1 2 3 4 5

Strongly Disagree Strongly Agree

1. My opinions about my current health status should be considered in my decision to be screened for prostate cancer.

1 2 3 4 5

Strongly Disagree Strongly Agree

1. My opinions about possible side effects of treatment for prostate cancer should be considered in my decision to be screened for prostate cancer.

1 2 3 4 5

Strongly Disagree Strongly Agree

Appendix S3

Patient Survey-Post

1. I have an OPTION to choose the PSA (prostate specific antigen) blood test to screen for prostate cancer.

1 2 3 4 5

Strongly Disagree Strongly Agree

1. There are factors in my personal or family history that may affect my risk for developing prostate cancer.

1 2 3 4 5

Strongly Disagree Strongly Agree

1. My opinions about my current health status should be considered in my decision to be screened for prostate cancer.

1 2 3 4 5

Strongly Disagree Strongly Agree

1. My opinions about possible side effects of treatment for prostate cancer should be considered in my decision to be screened for prostate cancer.

1 2 3 4 5

Strongly Disagree Strongly Agree

1. Please choose the 3 most important factors in your decision to accept or reject prostate cancer screening with the PSA test (check the 3 factors that apply).

_My age

_My race or ethnicity

_My family history of prostate cancer

_Previous or current physical exam

_Previous or current PSA test result

_My opinion about how healthy I am overall

_My concern about leakage of urine

_My concern about sex life

_My concern about developing prostate cancer

_My provider’s recommendation

Appendix S4

Provider Responses to Perceived Usefulness and Perceived Ease of Use scales


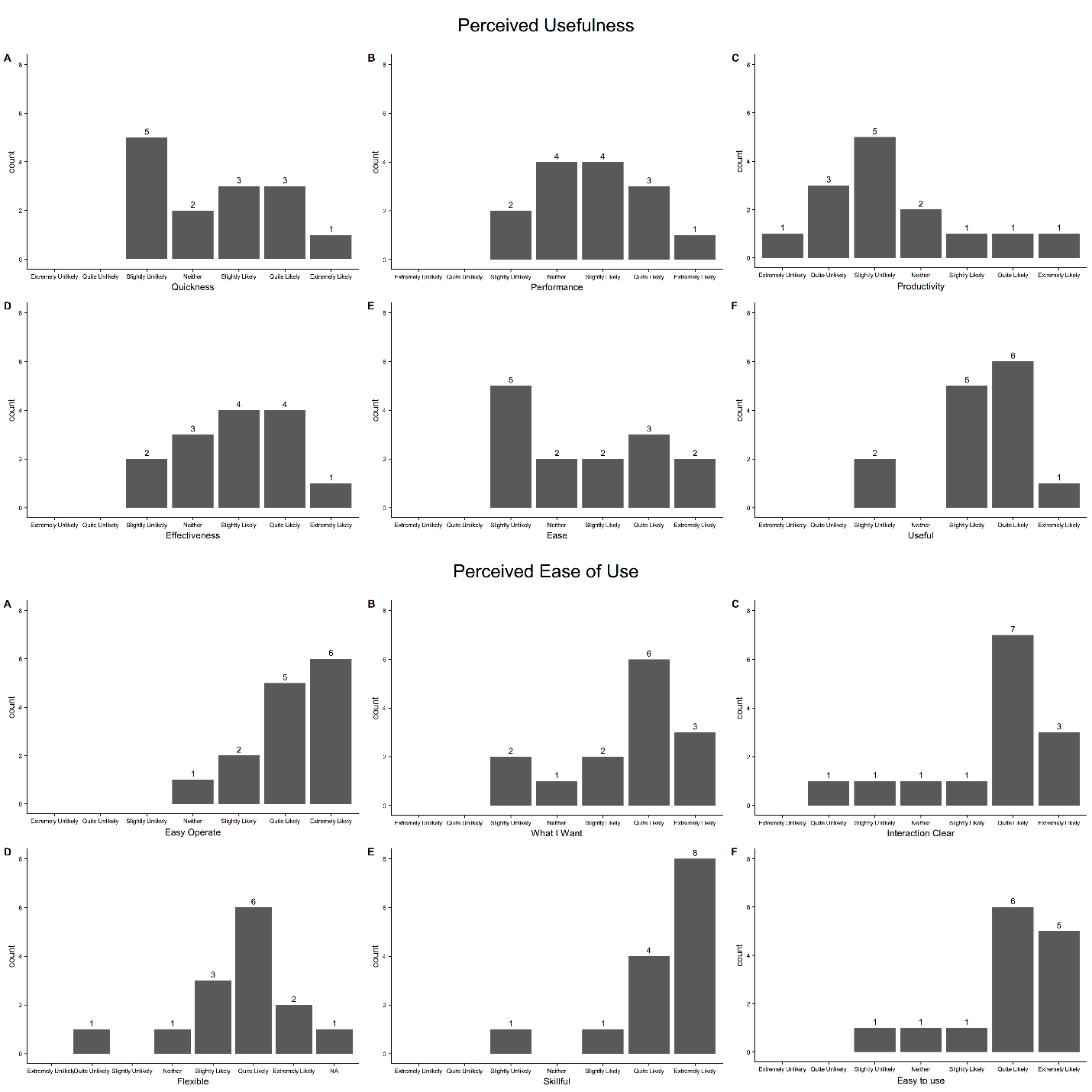

Supplement: Supplementary file 1 [file Data_Sheet_1.docx]
